# The “Real” Gibbs Paradox and a Composition-Based Resolution

**DOI:** 10.3390/e25060833

**Published:** 2023-05-23

**Authors:** Fabien Paillusson

**Affiliations:** School of Mathematics and Physics, University of Lincoln, Brayford Pool, Lincoln LN6 7TS, UK; fpaillusson@lincoln.ac.uk

**Keywords:** Gibbs paradox, mixtures, entropy

## Abstract

There is no documented evidence to suggest that J. W. Gibbs did not recognize the indistinguishable nature of states involving the permutation of identical particles or that he did not know how to justify on a priori grounds that the mixing entropy of two identical substances must be zero. However, there is documented evidence to suggest that Gibbs was puzzled by one of his theoretical findings, namely that the entropy change per particle would amount to $k_{B}\ln2$ when equal amounts of any two different substances are mixed, no matter how similar these substances may be, and would drop straight to zero as soon as they become exactly identical. The present paper is concerned with this latter version of the Gibbs paradox and, to this end, develops a theory characterising real finite-size mixtures as realisations sampled from a probability distribution over a measurable attribute of the constituents of the substances. In this view, two substances are identical, relative to this measurable attribute, if they have the same underlying probability distribution. This implies that two identical mixtures do not need to have identical finite-size realisations of their compositions. By averaging over composition realisations, it is found that (1) fixed composition mixtures behave as homogeneous single-component substances and (2) in the limit of a large system size, the entropy of mixing per particle shows a continuous variation from $k_{B}\ln2$ to 0, as two different substances are made more similar, thereby resolving the “real” Gibbs paradox.

## 1. Introduction

In most modern texts, the Gibbs paradox is referred to as the inability to ground the extensivity of thermodynamic potentials, such as the entropy or the Helmholtz free energy, within the framework of classical statistical mechanics developed by J. W. Gibbs in [1]. In his famous 1992 discussion of the Gibbs paradox, E. T. Jaynes summarises the situation well [2]:

“For 60 years, textbooks and teachers (including, regrettably, the present writer) have impressed upon students how remarkable it was that Gibbs, already in 1902, had been able to hit upon this paradox which foretold—and had its resolution only in—quantum theory with its lore about indistinguishable particles, Bose and Fermi statistics, etc.”

Jaynes, however, contends that Gibbs committed a mathematical mistake in his latest writings, which is ultimately responsible for the whole confusion around extensivity in statistical mechanics:

“In particular, Gibbs failed to point out that an “integration constant” was not an arbitrary constant, but an arbitrary function. However, this has, as we shall see, nontrivial physical consequences. What is remarkable is not that Gibbs should have failed to stress a fine mathematical point in almost the last words he wrote; but that for 80 years thereafter all textbook writers (except possibly Pauli) failed to see it.”

In the above passage, the mentioned integration constant would arise from a result derived by Gibbs in Chapter IV of his book on statistical mechanics. This point will be important for what we are going to discuss in a few paragraphs.

Whilst Jaynes’ intention may have principally been to celebrate Gibbs’ work and push against the necessity of a quantum foundation for classical statistical mechanics, these two quotes ironically end up further cementing a narrative that attributes a fault to Gibbs, which would have inadvertently led astray the physics and thermodynamics community for at least 80 years.

It is important to point out that such a narrative stems from a less-than-charitable, or even perhaps dishonest, reading of Gibbs’ writing in his final book on statistical mechanics [1]. Jaynes even ventured to suggest that Gibbs was *older* and in *bad health*, which explains why his latest piece of work was lacking compared to previous ones. Interestingly, even biographies written just a few years after Gibbs’ death, such as [3], do not appear to mention any purported intellectual decline or poor health in the later years of his life. However, one just needs to read the preface of *Elements of Statistical Mechanics* [1] to see that it was never Gibbs’ intention to consider systems with varying particle numbers; thus, any analysis of extensivity for the results presented in the preceding chapters is automatically excluded until Chapter XV, which is the final chapter of his book:

“Finally, in Chapter XV, we consider the modification of the preceding results which is necessary when we consider systems composed of a number of entirely similar particles of several kinds, all of which kind being entirely similar to each other, and when one of the numbers of variations to be considered is that of the numbers of the particles of the various kinds which are contained in the system.”

At this point, the reader may be reminded that the alleged “integration constant” mistake (conflating a function of the particle number with a constant) that Gibbs made, according to Jaynes, is in Chapter IV.

A full discussion of how both the aforementioned extensivity issue and the purported mistakes attributed to Gibbs are actually not supported by any evidence in Gibbs’ writings has been provided elsewhere [4]. In what follows, we shall assume that the few quotes provided above are sufficient to cast some suspicion about the historical, historiographical, and conceptual veracity of the main interpretation of Gibbs’ paradox found in most textbooks. We shall now safely depart from this view and delve into what we term as the “real” Gibbs’ paradox.

In his 1876 work *on the equilibrium of heterogeneous substances* [5], Gibbs found a somewhat intriguing result grounded in thermodynamics (but easily reproducible in statistical mechanics by later authors), where the change in entropy would amount to $k_{B}\ln2$ per unit of mass/matter upon mixing equal amounts of any two different non-reactive substances. The surprising fact was that this result held no matter how similar these substances would be. Here, again, we may directly quote Gibbs [5]:

“The fact is not less significant that the increase of entropy due to the mixture of gases of different kinds … is independent of the nature of the gases … and of the degree of similarity between them.”

If the substances were considered to be exactly identical, however, one would find the entropy change to be exactly zero.

In [6], it is reported that Pierre Duhem was likely the first to mention the paradoxical nature of this result and highlight the “absurd consequences” that violated the continuity principle. To our knowledge, very few textbooks on statistical mechanics actually refer to the latter when discussing the Gibbs paradox. The only two such texts we are aware of are [7,8].

Van Kampen proposed a discussion of this version of the Paradox in [9]. In this discussion, he proposes the following reasoning to dispel the notion that there is any paradox at all

“But suppose that [substances] A and B are so similar that the experimenter has no physical way of distinguishing between them. Then he does not have the semi-permeable walls needed for the second process, but on the other hand the first one will look perfectly reversible to him.”

This leads to the conclusion that there should not be any change in entropy as long as the experimenter does not have any physical mean (or is not interested in using any such means) to separate two substances. Indeed, in the above quote, the second process mentioned refers to obtaining the final entropy by determining the work performed by a semi-permeable membrane letting *A* pass through and not *B*, for example.

This statement by van Kampen on the operational interpretation of the entropy of mixing was recently demonstrated experimentally with colloids in [10], but had already been mentioned by Gibbs one hundred years earlier in [5]:

“when we say that two gas-masses of the same kind are mixed under similar circumstances there is no change of energy or entropy, we do not mean that the gases that have been mixed can be separated without change to external bodies. On the contrary, the separation of the gases is entirely impossible … because we do not recognise any difference in the substance of the two masses”,

and was also likely known by Duhem as well.

The author remains uncertain that van Kampen’s argument dispels the paradox pointed out by Gibbs and Duhem for all possible imaginable substances. For example, in his explanation, van Kampen assumes that either one can certainly discriminate between substance *A* and substance *B*, or one is incapable of discriminating them at all. However, what if, for example, substances *A* and *B* actually comprise the same molecules, but not in the same proportions? Surely a gas containing 80% dioxygen and 20% nitrogen is not “identical” to a gas containing 20% dioxygen and 80% nitrogen. How do we separate these two substances then, and what is the corresponding work needed to do so? As proposed in [4], it is possible to conceive of a generalised semi-permeable membrane that only has a given probability (less than or equal to unity) to bring one of the molecules of a given type from one side of the box to the other side, to separate substances *A* and *B*. A similar approach was used to experimentally test the Landauer bound in [11].

These new developments allow one to imagine *degrees of similarity* between substances rather than a binary identical/different view of substance similarity.

A further issue that arises when thinking about the mixing of real substances is well illustrated by a thought experiment discussed in [12]: If two glasses of milk are poured from the same bottle, they will correspond to two different finite realisations of the same underlying substance (i.e., the milk coming from the bottle). Consequently, the two glasses are unlikely to contain the very same amounts of, say, fat globules or casein proteins, despite the fact that they are sampled from the same underlying substance. If one were to mix these two glasses into a larger container, would one expect a non-zero mixing entropy from a statistical mechanics or thermodynamics perspective? More importantly, for the purpose of the present paper, how can the experiment be reproduced in the first place?

Before moving on to what the present article seeks to address, it will be instructive to look first at a common approach that has been extensively used in the past two decades to describe mixtures, discrete or otherwise, either in the context of the Gibbs paradox (e.g., in s [4,12,13]) or in the context of multimer assemblies [14] and phase equilibria of polydisperse systems (e.g., [15,16,17,18,19]). One way to conceive this approach consists in considering a mixture comprising a fixed integral number $N_{i}$ of particles of species *i* among, say, *m* species. If the system has a total of *N* particles, then we must have that $N=\sum_{i=1}^{m}N_{i}$. For an ideal gas model confined to a volume *V*, the corresponding free energy in the canonical ensemble is easily found to be in the large $N_{i}$ limit (see, e.g., [12])

(1) $$\beta F\left(N,\left\{N_{i}\right\},\beta \right)\approx -N\ln\frac{V}{\Lambda^{3}}-N+\sum_{i=1}^{m}N_{i}\ln N_{i},$$

where, for illustration purposes, we considered a gas of particles with no relevant internal degrees of freedom and possessing identical masses (thus distinguished by means other than their mass) and the thermal length scale Λ. The approach then goes on to make the following prescription:

(2) $$N_{i}=Np\left(i\right),$$

where p(i) is interpreted as being the probability of having a particle of species *i* in the system. Substituting the above prescription into Equation (1) gives

(3) $$F\left(N,\left\{N_{i}\right\},\beta \right)\approx Nk_{B}T\ln\left(n\Lambda^{3}\right)-N-Nk_{B}Ts\left(p\right),$$

where n=N/V is the particle number density and

(4) $$s\left(p\right)=-\sum_{i=1}^{m}p_{i}\ln p_{i}.$$

At this stage, it is worth pointing out that some disagreement exists in the naming convention used to qualify the quantity $k_{B}s\left(p\right)$ appearing in Equation (3) for the free energy of a single substance. For example, in [12], $k_{B}s\left(p\right)$ is called the *mixing entropy*, while in [13], $k_{B}s\left(p\right)$ is considered the characteristic of the composition of a substance (no matter how this substance is put together), as described by the probability p(i), and is, therefore, called *composition entropy*. Given that the present work is a continuation of the work proposed in [13], we shall adopt the latter naming convention in what follows. On that latter view, refs. [4,13] have defined the mixing entropy as the change in the composition entropy between a system comprising $N_{A}$ and $N_{B}$ particles of two initially separated substances, *A* and *B*, and that of a substance *C* resulting from putting *A* and *B* together in the same volume. If we denote $p_{A}$, $p_{B}$, and $p_{C}=\frac{N_{A}}{N}p_{A}+\frac{N_{B}}{N}p_{B}$ as the composition probabilities of substances *A*, *B*, and *C*, respectively, then the (dimensionless) entropy change is found to be [13]

(5) $$\Delta S/k_{B}=N_{A}D_{KL}\left(p_{A}|p_{C}\right)+N_{B}D_{KL}\left(p_{B}|p_{C}\right),$$

where

(6) $$KL\left(p|q\right)\equiv -\sum_{i=1}^{m}p\left(i\right)\ln\frac{q(i)}{p(i)}$$

is the Kullback–Leibler divergence of the probability *p* from probability *q*. In the context of strongly asymmetric mixing, e.g., $N_{A}\ll N_{B}$, it was shown in [13] that Equation (5) reduces to a prescription by Sollich et al. for determining the phase equilibria of polydisperse systems, for instance in [18,19], where a dominant (parent) composition starts coexisting with a minority (incipient) composition. The traditional Gibbs mixing thought experiment is, however, more in line with a fully symmetric case, where $N_{A}=N_{B}=N/2$. In this case, Equation (5) reduces to [4,13]

(7) $$\Delta S/k_{B}=ND_{JS}\left(p_{A}|p_{B}\right),$$

where

(8) $$D_{JS}\left(p_{A}|p_{B}\right)\equiv \frac{1}{2}\sum_{i=1}^{m}\left(p_{A}\left(i\right)\ln\left(\frac{2p_{A}\left(i\right)}{p_{A}\left(i\right)+p_{B}\left(i\right)}\right)+p_{B}\left(i\right)\ln\left(\frac{2p_{B}\left(i\right)}{p_{A}\left(i\right)+p_{B}\left(i\right)}\right)\right),$$

is the Jensen–Shannon divergence between $p_{A}$ and $p_{B}$, which corresponds to a square metric [20] between the probability distributions $p_{A}$ and $p_{B}$.

The problem with the above approach is that it entirely relies on the prescription that $N_{i}=Np\left(i\right)$. However, upon inspection, this prescription does not appear justified for three reasons:

- Firstly, from a mathematical standpoint, if a mixture is characterised by a probability distribution p(i), then each time a particle of that substance is added to the system, the species *i* is selected with probability p(i). In a simpler case, where only two species are possible, i.e., m=2, the problem becomes analogous to the flipping of *N* coins. In that case, the number of particles of a given species shall follow a binomial distribution, as studied in [21], and Np(i) is the (binomial) expectation value of $N_{i}$. In practice, however, a single realisation of *N* coin flips is not expected to give exactly $N_{i}=Np\left(i\right)$. Thus, the proposed prescription, albeit heuristically intuitive, conflates an instantiated value taken by a random variable with its expectation value; this is akin to a sort of ‘mean field’ approximation, especially given that $N_{i}$ ends up in various logarithmic functions in statistical thermodynamics.
- Secondly, from a conceptual standpoint, there is a problem with the substitution of $N_{i}$ by Np(i); the latter is often not an integer. Gibbs’ statistical mechanics, or its quantum extension, establishes a relationship between the dimensionality of state spaces to be explored and the number of particles in the system. These spaces possess an integral number of dimensions, not a fractional one.
- Finally, the prescription is often taken *after* the Stirling approximation has been used, which must assume that *each* $N_{i}$ is large; this cannot be guaranteed for all system sizes for all composition probabilities.

Despite the above critique of the $N_{i}=Np\left(i\right)$ prescription, Equation (7) ultimately appears to make sense from a physical standpoint, as discussed in [4,13], and it can be obtained from a substantial body of existing works [12,15,19]. It is just that (a) the prescription is not mathematically consistent with the probability theory and (b) it is not conceptually satisfactory. More particularly, there is no notion of what happens if *N* cannot be assumed to be infinite. The aim of the present paper is, therefore, to extend the work on binary mixtures carried out in [21] to generalised mixtures, so as to more firmly ground both mathematically and conceptually the validity of Equation (7), and propose mixing entropy expressions for finite *N*.

In what follows, we propose a new formalism that enables one to address the above questions. More specifically, we consider that substances are ultimately defined by composition probabilities and that one can only ever mix two finite composition realisations of different substances. Given that the entropy change will depend on the specific realisations one is looking at, the reproducibility requirement of thermodynamics compels us to seek realisation-independent mixing entropy expressions. We obtain the latter by averaging over composition realisations in both substances to be mixed. We believe that this approach follows the operational view promoted by Gibbs and van Kampen, but when the two substances cannot be separated with certainty and have some overlap in their composition.

## 2. Materials and Methods

The method we introduce in this paper expands upon a preliminary work by the present author on binary mixtures [21]. This generalisation can be formulated in the following presuppositions:

1- Discrete mixtures comprising *m* possible *identifiable species*, labeled from 1 to *m*, are characterised—in a definitional sense—by an *ideal composition* corresponding to a set of fixed probabilities ${\left\{p\left(i\right)\right\}}_{i=1,\ldots ,m}$, satisfying
  (9) $$\sum_{i=1}^{m}p\left(i\right)=1.$$
2- Any real mixture with a finite number *N* of particles is but one of many *realisations* obtained from *independently* sampling each *N* particle identity from the sample space {1,…,m}, with the corresponding probabilities ${\left\{p\left(i\right)\right\}}_{i=1,\ldots ,m}$.
3- Let $N_{i}$ denote the random variable representing the number of particles of species *i* in a given mixture and let $C=\{N_{1},\ldots ,N_{m}\}$ represent the multivariate random variable characterising the *empirical composition*. If the mixture comprises *N* particles. We must have that
  (10) $$\sum_{i=1}^{m}N_{i}=N.$$
4- We denote $C\equiv \{N_{1},\ldots ,N_{m}\}$ the *composition realisation* of a given mixture, such that for any species index *i*, we have $N_{i}=N_{i}$. Given that the indicator random variables for the *N* particles are considered independent and identically distributed with ${\left\{p\left(i\right)\right\}}_{i=1,\ldots ,m}$, it follows that the probability distribution for C satisfies a multinomial distribution:
  (11) $$M_{N,m,p}\left(C\right)=\frac{N!}{\prod_{i=1}^{m}N_{i}!}\prod_{j=1}^{m}p{\left(j\right)}^{n_{j}},$$
  where it is understood that $N_{i}$ must comply with Equation (10) as well.
5- The *Helmholtz free energy* $F(N,\beta =\frac{1}{k_{B}T},C)$ of an *N*-particle system is also a random variable via the composition random variable C. We seek the realisation-independent Helmholtz free energy of the system F(N,β) by averaging over all possible composition realisations:
  (12) $$F\left(N,\beta \right)\equiv \langle \langle F\left(N,\beta ,C\right)\rangle \rangle \equiv \sum_{C}M_{N,m,p}\left(C\right)F\left(N,\beta ,C\right).$$

To address Gibbs’ paradox with the above formalism, we also need to specify an ensemble and a model to apply it to. We choose to work in the Gibbs canonical ensemble, i.e., fixed temperature and a fixed amount of matter. Given that the paradox arises already in the case of ideal gases, the very first step is, therefore, to discuss it in the context of *N* non-interacting, non-relativistic, and independent particles confined in a box of volume *V*. This is what shall be done in the rest of this paper.

For a system of *N* particles comprising $N_{i}$ particles of species *i* of mass $M_{i}$ corresponding to a given composition realisation C, the Gibbs canonical partition function reads

(13) $$Z\left(N,\beta ,C\right)=V^{N}\prod_{i=1}^{m}\left(\frac{z_{i}^{N_{i}}}{N_{i}!\Lambda_{i}^{3N_{i}}}\right),$$

where $z_{i}$ is the internal dimensionless canonical partition function of a particle of type *i*, $\Lambda_{i}=\xi /\sqrt{2\pi M_{i}k_{B}T}$ is a characteristic length scale associated with the species *i*, and ξ is the area—preserved by the Hamiltonian flow—of an elementary two-dimensional face of a polygonal cell of a partition of the phase space. Of course, it is common in modern texts to identify $\Lambda_{i}$ to the thermal de Broglie wavelength by setting ξ=h, the Planck constant, but this is not a necessity.

From Equation (11), we have that

(14) $$\frac{1}{\prod_{i=1}^{m}N_{i}!}=\frac{M_{N,m,p}\left(C\right)}{N!\prod_{j=1}^{m}p{\left(j\right)}^{N_{j}}},$$

we find

(15) $$\beta F\left(N,\beta \right)=-NlnV+N\ln\left({\tilde{\Lambda }}^{3}/\tilde{z}\right)+\ln N!-Ns\left(p\right)+H\left(M_{N,m,p}|M_{N,m,p}\right),$$

where $\tilde{\Lambda }=e^{\sum_{i=1}^{m}p\left(i\right)\ln\Lambda_{i}}$ is a single effective characteristic length scale for the substance, $\tilde{z}=e^{\sum_{i=1}^{m}p\left(i\right)\ln z_{i}}$ is an effective internal partition function for the substance, $s\left(p\right)\equiv -\sum_{i=1}^{m}p\left(i\right)\ln p\left(i\right)$ is the composition entropy, $H\left(M_{N,m,p}|M_{N,m,p}\right)\equiv -\sum_{C}M_{N,m,p}\left(C\right)\ln M_{N,m,p}\left(C\right)$ is the *composition realisation entropy*, and we use the fact that $\langle \langle N_{i}\rangle \rangle =\sum_{C}P\left(C\right)N_{i}=Np\left(i\right)$ for the multinomial distribution (11).

One of the major difficulties in regard to using Equation (15) for analytical results for finite-sized systems is that the term $H(M_{N,m,p}|M_{N,m,p})$ corresponds to the entropy of the multinomial distribution and there is no known closed-form expression for it. We are, therefore, bound to explore different limiting regimes in the cases of different kinds of composition models, and propose conclusions on a case-by-case basis.

## 3. Results

Equation (15) is exact for any finite system consisting of *N* particles and characterised by a composition probability map *p*. This will serve as our starting point to elucidate the thermodynamic behaviours of general mixtures when they are considered on their own and also upon mixing any two mixtures. In particular, we shall also try to explore cases of so-called *continuous polydisperse* systems. In what follows, we shall use as a safeguarding strategy the principle that, in the large *N* limit, the realisation-independent free energy of mixture composition models should become extensive. Composition models that do not satisfy this requirement shall be considered provisionally inappropriate for the study of the thermodynamics of substances and would warrant further inspection outside of the scope of the present paper.

### 3.1. Case of a Single Mixture

As mentioned before, the potential utility of the proposed formalism is going to depend on one’s ability to evaluate the entropy of the multinomial distribution $H(P\left(C\right)|M_{N,m,p}\left(C\right))$. In what follows, we will use the following convenient rewriting of this entropy:

(16) $$H\left(M_{N,m,p}|M_{N,m,p}\right)=-\ln N!+Ns\left(p\right)+\sum_{i=1}^{m}\sum_{N_{i}=0}^{N}B_{N,p}\left(N_{i}=N_{i}\right)\ln\left(N_{i}!\right),$$

where $B_{N,p}\left(N_{i}=N_{i}\right)$ is the binomial probability distribution for finding $N_{i}=N_{i}$ particles of type *i* in the composition realisation given the specified *N* and p(i)

(17) $$B_{N,p}\left(N_{i}=N_{i}\right)\equiv \frac{N!}{\left(N-N_{i}\right)!N_{i}!}p{\left(i\right)}^{N_{i}}{\left(1-p\left(i\right)\right)}^{N-N_{i}}.$$

#### 3.1.1. Finite Discrete Mixtures: *m* and Map *p* Are Fixed and N>m

The case of finite discrete mixtures corresponds to the “typical” situation that most thermodynamicists might have in mind for a mixture, i.e., there is a clearly defined set of *m* different chemical species and various probabilities assigned to each species, and *N* is often much larger than *m*. If *N* is large enough, such that Np(i)⪆10 for any i=1,…,m, then one may use the Stirling approximation for $N_{i}!$ and take the normal distribution limit of $B_{N,p}\left(N_{i}=N_{i}\right)$ giving [22] (see also Appendix A for numerical comparisons)

(18) $$H\left(M_{N,m,p}|M_{N,m,p}\right)=\frac{m-1}{2}\ln\left(2\pi Ne\right)+\frac{1}{2}\sum_{i=1}^{m}\ln p\left(i\right)+O\left(\frac{1}{N}\right).$$

One notes that the first term in Equation (18) is of order ∼lnN, the second term is, at most, of order ∼lnm, and the remainder of order ∼1/N. This means that, in the large *N* limit, using the Stirling approximation, the realisation-free Helmholtz free energy per particle for finite discrete mixtures becomes

(19) $$\frac{F(N,T)}{N}≃k_{B}T(\ln\left(n_{v}{\tilde{\Lambda }}^{3}/\tilde{z}\right)-s\left(p\right)-1),$$

where we introduce the particle density $n_{v}\equiv N/V$. Three important remarks follow from Equations (18) and (19):

- At fixed composition, i.e., at fixed *m* and probability map *p* on {1,…,m}, s(p) is a constant, and the mixture can be essentially conceived as a homogeneous substance. All thermodynamic properties of the system will be identical to that of a single component system with only the total substance particle density $n_{v}$ (as opposed to partial densities) playing a role.
- In the large system size limit, the realisation-independent free energy becomes proportional to the total amount of matter *N* in the system, i.e., becomes extensive. Consequently, given the safeguarding criterion we chose, the finite discrete mixture model described above constitutes a valid composition model of general mixtures.
- By applying the defining relation for the thermodynamic entropy $S\left(N,T\right)\equiv -\frac{\partial F}{\partial T}$ to Equation (19), we obtain
  (20) $$S\left(N,T\right)≃Nk_{B}\ln V-\frac{3}{2}Nk_{B}\ln T+Nk_{B}T\frac{\partial \ln\tilde{z}}{\partial T}+K\left(p\right),$$
  where K(p) is a function that depends only on the composition characterised by the map *p*. This result for the entropy expression of a substance was already anticipated by Gibbs in 1876 in [5] in the case where $\tilde{z}=1$ (and with slightly different notations).

“[Equation (20)] applies to all gases of constant composition for which the matter is entirely determined by a single variable [*N*]”

We should note that Equation (19) has already been obtained by the author in [4], but within the heuristic theoretical framework described in the introduction section, whereby whole particle numbers for each species were directly replaced by their composition averages into the expression of the canonical partition function. This amounts to a sort of “mean-field” approximation, which is somewhat unjustified from a mathematical standpoint and has the conceptual disadvantage of invoking fractional particle numbers, which is hardly satisfactory.

On the contrary, the formalism proposed in Equations (9)–(15) leading to Equation (19) is free from these shortcomings.

#### 3.1.2. Infinite Discrete Mixtures: m>>N and $N^{2}p\left(i\right)\ll 1$

It is tempting to imagine that so-called continuous mixtures or continuous polydisperse mixtures would correspond to the composition model we term as *infinite discrete mixtures*, where m≫N, no matter the system size, including the thermodynamic limit, and where the values of all the probabilities p(i) are monotonously decreasing functions of *m* with zero as the asymptotic limit for $N^{2}p\left(i\right)$. A trivial example of this model would be the uniform probability model p(i)=1/m when $m\gg N^{2}\gg 1$.

For infinite discrete mixture models, obtaining a particle of a given species becomes incredibly rare and the number *N* of particles in the system is too small compared to *m* for any single realisation of the composition $C=\{N_{1},\ldots ,N_{m}\}$ to provide any faithful representation of the composition probabilities p(i). Consequently, the third term of Equation (16) is dominated by the value $N_{i}=2$ i.e.,

(21) $$\sum_{i=1}^{m}\sum_{N_{i}=0}^{N}B_{N,p}\left(N_{i}=N_{i}\right)\ln N_{i}!≃\frac{N(N-1)\ln2}{2}\sum_{i=1}^{m}p{\left(i\right)}^{2}∼O\left(N^{2}p\left(i\right)\right)\ll 1.$$

It follows that for infinite discrete mixtures, including cases where *m* tends to infinity at finite *N*, as well as in thermodynamic limit cases where the *m* limit is taken first, and only then the limit of infinite *N*, we obtain the entropy of multinomial distribution (see Appendix A for numerical comparisons)

(22) $$H\left(M_{N,m,p}|M_{N,m,p}\right)\approx -\ln N!+Ns\left(p\right),$$

which, upon being substituted in Equation (15), gives

(23) $$\beta F\left(N,\beta \right)\approx -N\ln V+N\ln\left({\tilde{\Lambda }}^{3}/\tilde{z}\right).$$

It is quite straightforward to see that Equation (23) does not provide an extensive form for the realisation-independent free energy F. Therefore, given our extensivity criterion aimed at ensuring consistency with thermodynamics, we shall consider the infinite discrete mixture model described in this sub-section as being inadequate to model a typical thermodynamic situation. In particular, it does not appear to capture the thermodynamics displayed by continuous polydisperse systems, such as colloids.

#### 3.1.3. Finite Continuous Mixtures: p(i)=ρ(i)Δ(i) and N>m

The inadequacy of the infinite continuous mixture model to characterise an expected thermodynamic behaviour of a single mixture stems from adopting a value for *m*, which is much larger than *N*. In that case, composition realisations with 0 or 1 particle per species are overwhelmingly more likely than those with at least one species having two particles. This pathological behaviour need not be rooted in the physics of the problem. Rather, it may arise from an inadequate categorisation of the different species in the system relative to the available number of particles *N*. In this situation, one may need to resort to a coarse-graining procedure, effectively grouping together different species into a much smaller number of effective particle species. With this in mind, we introduce *finite continuous mixture* composition models for which the probabilities p(i) can be expressed as p(i)=ρ(i)Δ(i) for any i=1,…,m, where ρ is the probability density of a continuous random variable *X* and where Δ(i) is an interval size of *X* associated to the species index value *i*. This is, in fact, a special case of finite discrete mixtures, where we require the intermediate probability density ρ to be a smooth function and to not wildly change as *m* is increased. In fact, in this model, we expect the experimental ρ to converge to some theoretical one as *m* is increased.

**Figure A2:**
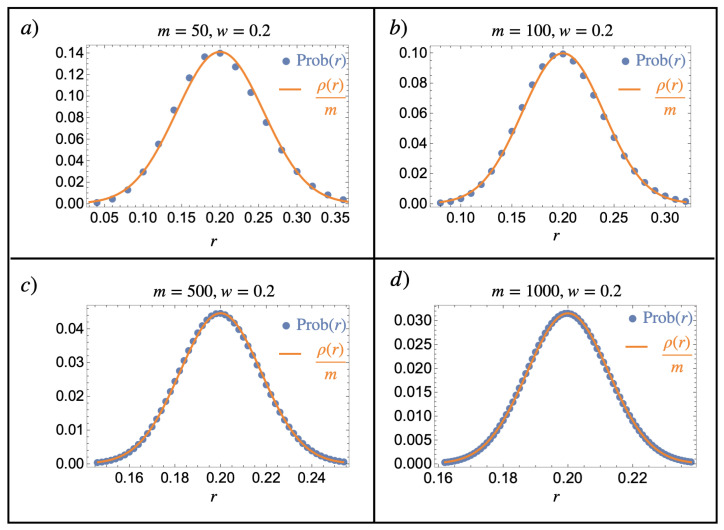
Normal limit of the binomial distribution. Comparison of Equations (24) (blue dots) and (26) (orange line) for various parameter values of *m* and w=0.2: (**a**) m=50, (**b**) m=100, (**c**) m=500, and (**d**) m=1000. We see that even for m=50, the normal distribution is already a good approximation of the binomial probability distribution, which becomes better as *m* is increased.

There are two distinct, but non-mutually exclusive, situations that would give rise to such models:

- Mathematical limit: The model for map p(i) may depend on *m* in a manner such that, as *m* becomes large “enough”, p(i) is very well-approximated by the distribution function of a continuous variable and is identical to it in the infinite *m* limit. Let us investigate in more detail the typical example of a binomial model for map p(i) with parameters *m* and *w*, i.e.,
  (24) $$p\left(i\right)=\frac{m!}{i!(m-i)!}w^{i}{\left(1-w\right)}^{m-i},$$
  where *w* is a new parameter characteristic of the composition model. Within this model, the most probable type of particle is that with the integer value, the closest to mw. Any particle index i≠mw will have a lower probability that decays rapidly to approach very small values as *m* becomes large enough. Indeed, in the large *m* limit at a fixed *w*, we have that the binomial distribution tends to a normal distribution (see Appendix B for a numerical comparison)
  (25) $$p\left(i\right)≃\frac{e^{-\frac{{\left(i-mw\right)}^{2}}{2mw(1-w)}}}{\sqrt{2\pi mw(1-w)}}.$$
  Consider now the map between the species label *i* taking values in {1,…,m} and a new variable r≡i/m taking values in the finite interval of the rationals {1/m,…,1}. From Equation (25) and the bijective character between *i* and *r*, it follows that
  (26) $$\mathrm{Prob}\left(r\right)≃\frac{e^{-\frac{{\left(r-w\right)}^{2}}{2w(1-w)/m}}}{\sqrt{2\pi w(1-w)/m}}\frac{1}{m}.$$
  In expression (26), *r* must be limited to take values in the set {1/m,…,1}. However, we really do have that Prob(r)=ρ(r)Δ(r), where ρ(x) is the probability density function of a normally distributed *continuous* random variable, and where we can identify Δ(r)=1/m. (cf. Figure A2 in Appendix B).
- Experimental considerations: Whether the composition is characterised by the preparation protocol or by a measurement technique, any experimental process is accompanied by a corresponding finite precision. Thus, in practice, even if one were to use a particle attribute that takes continuous values and is associated with a theoretical underlying ρ to characterise the composition of a mixture, it is bound to be expressed in terms of a discrete set {Δ(1),…,Δ(m)} of a finite number *m* of continuous intervals effectively corresponding to the *m* identifiable ’species’ of the system with the provided resolution and particle number. If *m* were to be increased, this would just improve the “granularity” of these intervals without making the set actually continuous. If this granularity is fine enough, one may *define* an empirical probability density $\rho_{\mathrm{emp}}\left(i\right)\equiv p\left(i\right)/\Delta \left(i\right)$ and fit it to a corresponding continuous probability density model. Convergence to a stable continuous model is expected as *m* (i.e., the resolution) is increased. This experimental resolution aspect can also be justified under the assumption that the excess free energy of the mixture solely depends on a subset of moments of the polydisperse distribution [19], where these moments are related to the precision of the measurement.

Regardless of whether the use of a continuous random variable and its associated probability density is justified by a mathematical limit of the composition model or inevitable experimental precision considerations, or both, ultimately, these preconditions allow one to write p(i)=ρ(i)Δ(i), where ρ is the probability density of a continuous random variable and for which N>m.

Given that N>m, we can use the approximate expression for the entropy of the multinomial distribution from Equation (18) and apply it to finite continuous mixtures. Substituting it into Equation (15), we obtain the following result in the limit of large *N*:

(27) $$\frac{F(N,T)}{N}≃k_{B}T\left(\ln\left(n_{v}{\tilde{\Lambda }}^{3}/\tilde{z}\right)-s_{\Delta }\left(\rho \right)-1\right),$$

where we use the Taylor–McLaurin formula to the first order to replace the discrete sum for the composition entropy s(p) by $s_{\Delta }\left(\rho \right)\equiv -\int_{-\infty }^{+\infty }\rho \left(x\right)\ln\left(\rho \left(x\right)\Delta \left(x\right)\right)dx$.

We note from Equation (27) that F is extensive and, thus, finite continuous mixtures serve as valid composition models for probing the thermodynamic behaviors of general mixtures in finite and large *N* system sizes. This is not too surprising given that, as stated above, what we call finite continuous models are special cases of finite discrete models with some additional regularity and smoothness requirements. Therefore, Equation (20) would hold as well for such mixture models.

### 3.2. Mixing of Two Substances

We will now utilize the results obtained in the previous section to calculate the entropy change that occurs when two finite discrete mixtures, substances *A* and *B*, are mixed. We denote *m* the number of distinct possible chemical species in substances *A* and *B* so that all particle types of substances *A* and *B* have a label *i*, taking values in the same sample space {1,…,m}. We denote $p_{A}\left(i\right)$ (resp. $p_{B}\left(i\right)$) as the probability for substance *A* (resp. *B*) to have a particle of type *i*.

As is common in the literature, we consider a mixing scenario comprising two different equilibrium situations:

- Firstly, let us consider a situation where a box of volume *V* is separated into two equally sized compartments by a removable wall with N/2 particles of substance *A* in, say, the left-hand side compartment, and N/2 particles of substance *B* in the right-hand-side compartment.
- Secondly, the wall separating the substances is removed so as to let them intermix until equilibrium is reached.

This scenario is the usual one within which the Gibbs paradox of mixing tends to be discussed. The quantitative aspects may be affected if one is not mixing an equal amount of substances *A* and *B*, or further contributions to the entropy changes can be expected if the densities in the two compartments are not initially the same.

#### 3.2.1. Free Energy before Removing the Wall

Before the wall is removed, each substance is separated into its own compartment and at equilibrium at the same temperature. Introducing the composition of multivariate random variables $C_{A}$ and $C_{B}$, the free energy in each compartment is also a random variable, which is a function of either $C_{A}$ or $C_{B}$, which, upon averaging over composition realisations in each compartment, gives

(28) $$\beta F_{A/B}\left(\frac{N}{2},\beta \right)=\frac{N}{2}\left(\ln\left(\frac{{\tilde{\Lambda }}_{A/B}^{3}}{{\tilde{z}}_{A/B}}\right)-\ln\frac{V}{2}-s\left(p_{A/B}\right)\right)+\ln\left(\frac{N}{2}!\right)+H\left(M_{\frac{N}{2},m,p_{A/B}}|M_{\frac{N}{2},m,p_{A/B}}\right),$$

where we have simply reproduced Equation (15) with indices *A* or *B* to specify whether the substance being looked at is characterised by $p_{A}$ or $p_{B}$.

In the end, from the additivity of the free energy, we obtain that

(29) $$F_{\mathrm{unmix}}\left(N,\beta \right)\equiv F_{A}\left(\frac{N}{2},\beta \right)+F_{B}\left(\frac{N}{2},\beta \right).$$

#### 3.2.2. Free Energy after Having Removed the Wall

Once the separating wall has been removed, substances *A* and *B* will mix and eventually reach equilibrium. After mixing, the number of particles of type *i* is going to be associated with the random variable $N_{i}^{\mathrm{mix}}=N_{i}^{A}+N_{i}^{B}$, where $N_{i}^{A/B}$ is the random variable corresponding to the number of particles of type *i* in substance *A* (resp. *B*). Given that each substance composition follows a multinomial distribution $M_{\frac{N}{2},m,p_{A/B}}$, it follows that

(30) $$\langle \langle N_{i}^{\mathrm{mix}}\rangle \rangle =\frac{N}{2}\left(p_{A}\left(i\right)+p_{B}\left(i\right)\right)\equiv Np_{\mathrm{mix}}\left(i\right),$$

where we identify $p_{\mathrm{mix}}$ the ideal composition probability distribution of the mixture of *A* and *B*. In Equation (30), the averaging over composition realisations is done by using the joint probability measure $\mathrm{Prob}\left(C_{A},C_{B}\right)=M_{\frac{N}{2},m,p_{A}}\left(C_{A}\right)M_{\frac{N}{2},m,p_{B}}\left(C_{B}\right)$, where the substances are considered completely independent.

The repeated mixing of N/2 particles from different composition realisations of substances *A* and *B* can be conceived as an actual protocol to sample *N* particles from the effective composition $p_{\mathrm{mix}}$. The probability of obtaining a given composition $C_{\mathrm{mix}}=\left\{N_{1}^{\mathrm{mix}},\ldots ,N_{m}^{\mathrm{mix}}\right\}$ then follows the multinomial distribution

(31) $$M_{N,m,p_{\mathrm{mix}}}\left(C_{\mathrm{mix}}\right)=\frac{N!}{\prod_{i=1}^{m}N_{i}^{\mathrm{mix}}!}\prod_{j=1}^{m}p_{\mathrm{mix}}^{N_{i}^{\mathrm{mix}}}=\frac{N!}{\prod_{i=1}^{m}\left(N_{i}^{A}+N_{i}^{B}\right)!}\prod_{j=1}^{m}p_{\mathrm{mix}}^{N_{j}^{A}+N_{j}^{B}},$$

where the last equality makes the connection between $N^{\mathrm{mix}}$ and $N^{A/B}$ stemming from the aforementioned mixing protocol used to sample $p_{\mathrm{mix}}$.

For two given composition realisations, $C_{A}$ and $C_{B}$, each comprising N/2 particles, we also have that, after mixing, the canonical partition function reads

(32) $$Z_{\mathrm{mix}}\left(N,\beta ,C_{A},C_{B}\right)=\frac{V^{N}}{\prod_{i=1}^{m}\left(N_{i}^{A}+N_{i}^{B}\right)!}\prod_{j=1}^{m}{\left(\frac{z_{j}}{\Lambda_{j}^{3}}\right)}^{N_{j}^{A}+N_{j}^{B}}.$$

Substituting Equation (31) into Equation (32), we have

(33) $$Z_{\mathrm{mix}}\left(N\beta ,C_{A},C_{B}\right)=\frac{V^{N}M_{N,m,p_{\mathrm{mix}}}\left(C_{A}+C_{B}\right)}{N!\;\prod_{i=1}^{m}p_{\mathrm{mix}}^{N_{i}^{A}+N_{i}^{B}}}\prod_{j=1}^{m}{\left(\frac{z_{j}}{\Lambda_{j}^{3}}\right)}^{N_{j}^{A}+N_{j}^{B}},$$

where $C_{A}+C_{B}=\left\{N_{1}^{A}+N_{1}^{B},\ldots ,N_{m}^{A}+N_{m}^{B}\right\}$.

In the end, upon averaging over composition realisations $C_{A}$ and $C_{B}$, we have

$$\begin{array}{cc} \beta F_{\mathrm{mix}}\left(N,\beta \right)= & -N\ln V+\ln N!-Ns\left(p_{\mathrm{mix}}\right)+\frac{N}{2}\ln\left(\frac{{\tilde{\Lambda }}_{A}^{3}{\tilde{\Lambda }}_{B}^{3}}{{\tilde{z}}_{A}^{3}{\tilde{z}}_{B}^{3}}\right) \end{array}$$

(34) $$\begin{array}{c} +H(M_{\frac{N}{2},m,p_{A}}M_{\frac{N}{2},m,p_{B}}|M_{N,m,p_{\mathrm{mix}}}), \end{array}$$

where it is understood that the last term involves sums over values taken by $N_{i}^{A}$ and $N_{i}^{B}$.

#### 3.2.3. Entropy Change

Given that the energy in the system does not change upon mixing (because the species are non-reactive and non-interacting), we now define the *entropy change upon mixing* as being $\Delta S_{\mathrm{mix}}\equiv \left(-F_{\mathrm{mix}}+F_{\mathrm{unmix}}\right)/T$, which gives:

(35) $$\Delta S_{\mathrm{mix}}\left(N,\beta \right)=k_{B}\ln\left(\frac{2^{N}{\left(N/2!\right)}^{2}}{N!}\right)+Nk_{B}D_{JS}\left(p_{A}|p_{B}\right)+k_{B}\Delta H,$$

where $\Delta H=-H\left(M_{\frac{N}{2},m,p_{A}}M_{\frac{N}{2},m,p_{B}}|M_{N,m,p_{\mathrm{mix}}}\right)+\sum_{i=A,B}H\left(M_{\frac{N}{2},m,p_{i}}|M_{\frac{N}{2},m,p_{i}}\right)$ and where $D_{JS}\left(p_{A}|p_{B}\right)$ is the Jensen–Shannon divergence introduced in Equation (8).

Equation (35) constitutes the main result of this paper and gives an *exact* realisation-independent expression of the classical mixing entropy of equal amounts of any two ideal-gas-like mixtures, *A* and *B*, for any system size *N*. A similar expression has already been found for binary mixtures in [21], but its generalisation to any extensive mixture model is, to our knowledge, new. It is worth going through the meaning of each of the terms in the entropy change expression.

- $\ln\left(\frac{2^{N}{\left(N/2!\right)}^{2}}{N!}\right)$ corresponds to the *partitioning entropy*, i.e., the entropy gained by releasing initially confined particles into double the initial volume. Note that this term is oblivious to the particle type and is, therefore, always positive, even if the substances are identical.
- $D_{JS}\left(p_{A}|p_{B}\right)$ measures a specific, bounded, square distance between substances $p_{A}$ and $p_{B}$. We shall propose an additional interpretation for this term a bit later.
- ΔH represents the entropy change owing to composition realisations, i.e., to the fact that, upon repeating experiments, one is bound to have different sampled empirical compositions from the ideal compositions expressed by the probability distributions $p_{A}$ and $p_{B}$.

Unfortunately, there is no closed-form expression for ΔH, which involves the entropy and cross-entropy of multinomial distributions. In practice, each of these terms in ΔH needs to be evaluated numerically for given composition models. For the large *N*, however, it can be shown (cf. Appendix C) that ΔH∼O(lnN).

For the large *N*, we also have that the first term in Equation (35) is actually of the form $∼\ln\left({\left({\frac{N}{2}}^{N/2}\right)}^{2}2^{N}N^{-N}\right)+O\left(\ln N\right)∼O\left(\ln N\right)$. Consequently, for the large *N*, i.e., for the system sizes initially considered by Gibbs, the only contribution (which happens to be extensive) left for the entropy of mixing is

(36) $$\Delta S_{\mathrm{mix}}^{\mathrm{Gibbs}}=Nk_{B}D_{JS}\left(p_{A}|p_{B}\right).$$

**Figure 1:**
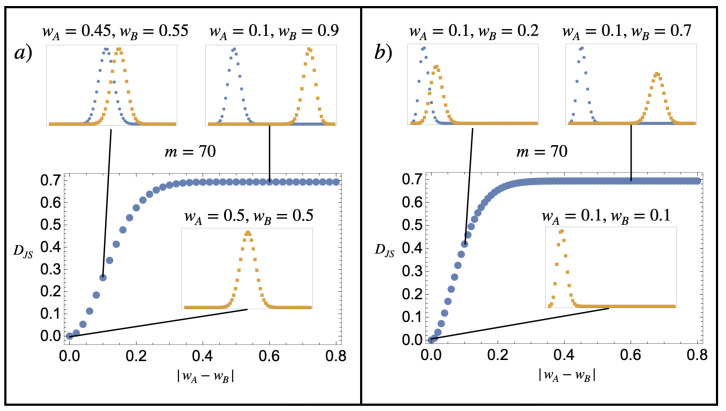
*Dimensionless Gibbs mixing entropy per particle*. The main plots represent $D_{JS}\left(p_{A},p_{B}\right)$ for two probability distributions $p_{A}$ (blue) and $p_{B}$ (orange), which follow the composition model of Equation (24), with m=70 and various values of $w_{A}$ and $w_{B}$ indicated in the figure. The similarity between the distribution is characterised by the absolute value in the difference between the *w* parameters $|w_{A}-w_{B}|$. When it is zero (the probability graphs are on top of one another), the substances are identical in the sense that they have the same composition probabilities. The figures show that the dimensionless Gibbs mixing entropy per particle varies continuously from 0 to ln2≈0.69 as the similarity between the compositions $p_{A}$ and $p_{B}$ is decreased. The exact details of how it does so depend on how $|w_{A}-w_{B}|$ is varied. (**a**) Having both distributions moving closer to w=0.5 in a symmetric fashion and (**b**) fixing $w_{A}=0.1$, with $w_{B}$ varying from 0.1 to 0.9.

Note that the Jensen–Shannon divergence $D_{JS}\left(p_{A}|p_{B}\right)$ is positive definite and is bounded from above by ln2. Figure 1 illustrates the value of the Gibbs entropy of mixing for two substances, *A* and *B*, corresponding to a finite discrete model of the kind described in Equation (24). Contrary to the sentence by Gibbs quoted in Section 1, we see that the Gibbs mixing entropy varies *continuously* from ln2 to 0 as the substance compositions become more similar.

## 4. Discussion

In 1876, Gibbs derived results that are generally undisputed; he showed that the mixing entropy of two substances would amount to $k_{B}\ln2$ per particle, irrespective of the degree of similarity between the substances, but would discontinuously drop to 0 as soon as they were *sensibly* exactly identical.

In this article, we have developed an approach to address the thermodynamic behaviour of general mixtures. This approach relies on the characterisation of the composition of a substance by an *a priori* probability distribution *p* acting on the space of all identifiable species. For a given composition probability *p*, any real system can just sample from that distribution and provide finite-size realisations of it. Thermodynamic quantities are retrieved from averaging over composition realisations of the Helmholtz free energy, the Massieu potential for fixed temperature, and system size ensembles. We found that a restricted class of composition models—that of finite discrete mixtures—was suitable for describing the extensive behaviour of a given substance. These models also encompass the possibility of characterising the particle attribute with a continuous variable. On the contrary, it was found that attempting to characterise a continuous mixture by setting the number of particle types to be much larger than the number of particles *N* in the system was inconsistent with thermodynamics. One intuitive explanation of this failure is that such infinite continuous mixture models do not allow for any finite-size empirical realisation of size *N* to actually be representative of the underlying distribution they are sampled from, to the extent that they all appear to be completely different, and therefore incommensurate with, any of the other realisations. It must be noted that using intervals of a continuous random variable to group many species into a similar effective species is not the only way of dealing with the thermodynamically invalid behaviour of what we call infinite continuous mixture models. The crucial aspect is mostly to reduce the dimensionality of the space within which one characterises the composition. This can also be done via a dimensional reduction approach that relies on determining a few moments of the underlying probability distribution of a specific attribute. The readers interested in such an approach may look at the work developed in [18,19], for instance.

For the selected class of models, it was found that, at a fixed composition, the thermodynamic behaviour is indistinguishable from that of a homogeneous one-component system, as already anticipated by Gibbs in the 1870s.

Applying this new formalism to a mixing scenario of equal amounts of two different substances, a general exact expression was derived for the classical mixing entropy of ideal substances. It was found that (a) even in cases where the substances were identical (same underlying probability distribution), some contributions to the mixing entropy would be non-zero, namely the partitioning entropy and the composition realisation entropy, and (b) in the large system size limit, this exact expression converges to a universal quantity that we call the Gibbs mixing entropy, which is extensive, and proportional to the square distance between the two ideal probability distributions characterising the substances being mixed. This extensive entropy of mixing was shown to vary continuously from 0 to $k_{B}\ln2$ per particle as the degree of dissimilarity was increased between the substances. This allowed us to provide a resolution to the paradoxical violation of the continuity principle pointed out by Duhem in the finding of Gibbs.

There are various open questions left to answer with regard to the view developed in the present paper and which will be left for future works: (1) while it is mostly straightforward to extend the proposed formalism to the semi-classical limit of quantum statistical mechanics of independent particles (essentially replacing discrete sums by integrals), it is much less obvious to the author as to how to implement it for quantum statistical situations, where the fermionic or bosonic characters of the particles matter. By this, we mean that, as discussed in [4], it is unclear how to make the fermion/boson character of elementary and composite particles compatible with both the existing body of works on polydisperse systems and the present one. (2) Many real substances are also (at least partially) reactive substances and, therefore, a given particle may not maintain a specific identity at all times. Some stationary compositions may be obtained but, in some systems with reactive processes occurring over time scales larger than thermodynamic experiments, some ergodicity considerations with regard to the present approach may be warranted.

In conclusion, in this paper, we have shown that what we have referred to as “real” Gibbs paradox could find a resolution within classical statistical mechanics by employing a composition-based description of general substances and examining averages over finite-size composition realisations.

## Figures and Tables

**Figure A1:**
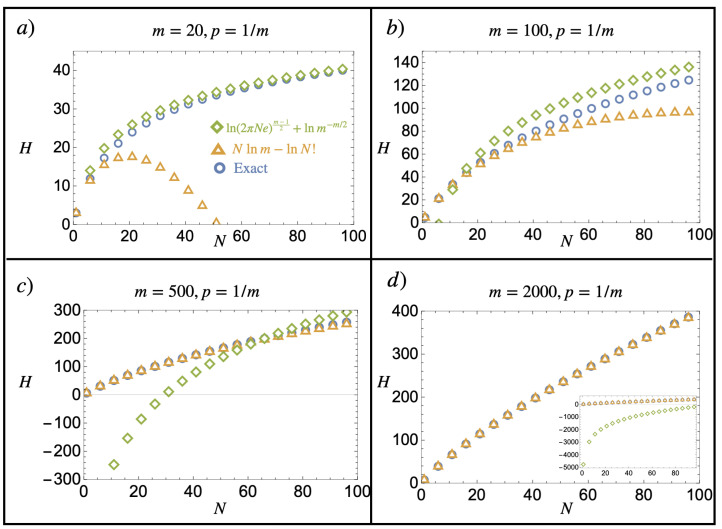
Entropy of the binomial distribution. Numerical comparison of the exact entropy of the binomial distribution (blue open circles) with Equation (18) (green open diamonds) and Equation (22) (orange open triangles), focusing on a uniform composition model, where p(i)=1/m, and exploring various values of *m* and a function of *N*. (**a**) m=20 shows good agreement between Equation (18) and the exact entropy from N⪆60≃3m. (**b**) For the case of m=100 and N≤100, neither Equation (18) nor Equation (22) provide a good approximation to the exact entropy. However, both expressions appear to provide reliable upper and lower bounds, respectively. (**c**,**d**) When the value of *m* is larger than *N*, Equation (22) is a good approximation for the entropy of the multinomial distribution. In (**c**), the deviation from good fit starts to become apparent when N≃100≃m/5.

## Data Availability

Data are contained within the article.

## Appendix A

This section focuses on numerical testing to determine the extent to which Equations (18) and (22) can serve as useful approximate expressions for the entropy of the multinomial distribution $H(M_{N,m,p}|M_{N,m,p})$. In Figure A1, we test it on a simple uniform composition model, i.e., p(i)=1/m, and propose various cases that depend on the values of *m* and *N*.

## Appendix B

This section illustrates the notorious normal distribution limit of the binomial distribution in the context of a composition model following Equation (24). In particular, it shows the main idea underlying the mathematical justification of the introduced notion of finite continuous mixtures by comparing Equations (24) and (26). A numerical implementation of different values of *m* and *w* is given in Figure A2.

## Appendix C

Given that there is no closed-form expression for ΔH in Equation (35), we are bound to find limiting cases for relatively large *N* values. This can be done by exploiting a few properties of ΔH:

- From Jensen’s inequality [23] 〈〈f(X)〉〉≥〈〈f(X)〉〉, it follows that, by choosing f(x)=lnx, and
  (A1) $$X=\frac{M_{N,m,p_{\mathrm{mix}}}\left(C_{A}+C_{B}\right)}{M_{\frac{N}{2},m,p_{A}}\left(C_{A}\right)M_{\frac{N}{2},m,p_{B}}\left(C_{B}\right)},$$
  we have
  (A2) $$\begin{array}{cc} -H(M_{\frac{N}{2},m,p_{A}}M_{\frac{N}{2},m,p_{B}}|M_{N,m,p_{\mathrm{mix}}}) & +H(M_{\frac{N}{2},m,p_{A}}M_{\frac{N}{2},m,p_{B}}|M_{\frac{N}{2},m,p_{A}}M_{\frac{N}{2},m,p_{B}})\geq 0\\ \; & ∴\Delta H\geq 0 \end{array}$$
- It is possible to estimate the order of magnitude of ΔH in the large *N* limit. Indeed, given that the entropy of a multinomial distribution is positive, it automatically follows that
  (A3) $$\begin{array}{cc} \Delta H & \leq H\left(M_{\frac{N}{2},m,p_{A}}M_{\frac{N}{2},m,p_{B}}|M_{N,m,p_{\mathrm{mix}}}\right)+\sum_{i=A,B}H\left(M_{\frac{N}{2},m,p_{i}}|M_{\frac{N}{2},m,p_{i}}\right)\\ \; & \leq 2\sum_{i=A,B}H\left(M_{\frac{N}{2},m,p_{i}}|M_{\frac{N}{2},m,p_{i}}\right), \end{array}$$
  where we use Equation (A2) and the fact that $H(M_{\frac{N}{2},m,p_{A}}M_{\frac{N}{2},m,p_{B}}|M_{\frac{N}{2},m,p_{A}}M_{\frac{N}{2},m,p_{B}})$$=\sum_{i=A,B}H\left(M_{\frac{N}{2},m,p_{i}}|M_{\frac{N}{2},m,p_{i}}\right)$ for the latter inequality. From Equation (18), it follows that ΔH⪅O(lnN) for large *N*.
